# Quantum stochastic walks on networks for decision-making

**DOI:** 10.1038/srep23812

**Published:** 2016-03-31

**Authors:** Ismael Martínez-Martínez, Eduardo Sánchez-Burillo

**Affiliations:** 1 Düsseldorf Institute for Competition Economics (DICE), Heinrich Heine Universität Düsseldorf, Universitätsstraße 1, D-40225 Düsseldorf, Germany; 2 Instituto de Ciencia de Materiales de Aragón (ICMA), CSIC-Universidad de Zaragoza and Departamento de Física de la Materia Condensada, Universidad de Zaragoza, E-50009 Zaragoza, Spain

## Abstract

Recent experiments report violations of the classical law of total probability and incompatibility of certain mental representations when humans process and react to information. Evidence shows promise of a more general quantum theory providing a better explanation of the dynamics and structure of real decision-making processes than classical probability theory. Inspired by this, we show how the behavioral choice-probabilities can arise as the unique stationary distribution of quantum stochastic walkers on the classical network defined from Luce’s response probabilities. This work is relevant because (i) we provide a very general framework integrating the positive characteristics of both quantum and classical approaches previously in confrontation, and (ii) we define a cognitive network which can be used to bring other connectivist approaches to decision-making into the quantum stochastic realm. We model the decision-maker as an open system in contact with her surrounding environment, and the time-length of the decision-making process reveals to be also a measure of the process’ degree of interplay between the unitary and irreversible dynamics. Implementing quantum coherence on classical networks may be a door to better integrate human-like reasoning biases in stochastic models for decision-making.

Systematic violations of assumptions in the standard model for rationality were reported by Tversky and Kahneman^1^ with great impact^2^,^3^. Numerous empirical findings in cognitive psychology and behavioral sciences exhibit anomalies with respect to the classical benchmark defined by the Kolmogorovian probability axioms and the rules of Boolean logic, suggesting a whole new scope of research: the development of models for decision-making based on the quantum formulation of probability theory^4^,^5^,^6^,^7^,^8^. The similarity between the epistemological content of the quantum theory and the situation in philosophy and psychology, where there is a vague separation between the subject and the object under study, was already at the heart of the seminal debate on the notion of complementarity^9^,^10^,^11^. Of course, we are not proposing any quantum physical implication for brain physiology, which remains as a controversial debate^12^,^13^.

We are interested in the potential of using the quantum formulation of probability theory into modelling decision-making schemes capable of integrating those behavioral aspects which the classical and normative paradigm of rationality needs to describe as mistakes^14^. The use of certain mathematical tools of quantum probability theory for the description of macroscopic systems usually thought as ‘just classical’ is a growing field of research^15^. In particular, and concerning the context of decision-making, beim Graben and Atmanspacher^16^ show that quantum statistics can arise from neural systems, even though the original model is not quantum but classical.

When applied to modelling cognition, the quantum-based model makes a universal, non-parametric prediction for the presence of order effects in attitude judgments which have been observed, for example, in large-scale American representative surveys^17^. Recently, Yearsley and Pothos^18^ defined a test for violations of the Leggett-Garg inequalities (temporal-Bell) in order to falsify the so-called ‘cognitive realism’ hypothesis. As the authors explain, “the observation of such a violation would indicate a failure of the top-down approach to cognition, in a classical, realist way”. Their standpoint is closely related to the proposal by Atmanspacher and Filk^19^ for the study of bistable perception. Kvam *et al.*^20^ demonstrate how deliberation modelled as a constructive process for evidence accumulation is better described with a quantum model than with the popular classical random walk.

Within the domain of economics and game theory, the link between Bell’s notion of nonlocality and Harsanyi’s theory of games with incomplete information has been object of novel attention^21^, as well as the comparison between the effects of classical and quantum signalling in games^22^,^23^,^24^. Concerning the crucial Aumann’s agreement theorem^25^, Khrennikov and Basieva^26^ show how agents using a quantum probability system for decision-making can indeed agree to disagree even if they have common priors, and their posteriors for a given event are common knowledge. In addition, Lambert-Mogiliansky *et al.*^27^ show how violations of transitivity of preferences in observed choices emerge naturally when dealing with non-classical agents, in line with the works by Makowski *et al.*^28^,^29^ who analyze how an agent achieves the optimal outcome through a sequence of intransitive choices in a quantum-like context.

As a consequence of the nature of the cognitive processes being better explained from the quantum probabilistic (or logic) viewpoint, Busemeyer *et al.*^30^,^31^ propose a quantum dynamic model of decision-making, as opposed to the Markovian settings previously established. Asano *et al.*^32^,^33^ elaborate deeper on the representability of these effects by understanding the decision-maker as a quantum open system, with the dynamics of the global system driven by the quantum analogy of the master equation.

Inspired by these latter developments, we propose a way to reconcile the novel application of quantum techniques with the classical origin of the problem of understanding human decision-making. Once that we accept the need for a non-classical extension of the standard models for decision-making, this paper addresses the question of *“how we can model the deliberation process generating the behavioral probabilities in a quantum manner”*.

We show how a quantum stochastic evolution of the relevant decision-making variables can be defined in terms of a linear superoperator deeply rooted in two fundamental elements of classical decision theory: (i) the ability of the decision-maker to discriminate between the available options, and (ii) the process of formation of beliefs in situations of uncertainty. Besides, the relaxation time reveals to be driven by the level of interpolation between the purely quantum and the purely classical random walk, in addition to a tradeoff with the relative weight the decision-maker assigns between the comparison of alternatives’ profitabilities and the formation of expectations on the possible states of the world.

The paper is organized as follows. We first define the evolution of the cognitive state as a case of quantum stochastic walks, and show how the dynamics of the walker can be represented by a network. We obtain the cognitive network as a natural extension of the well established decision-making trees, relying on the classical probabilistic choice model. Finally, we illustrate the class of quantum stochastic walks on networks for decision-making with the famous example of the Prisoner’s Dilemma game, a task which implies situations of strategic uncertainty. We provide a Methods section with rigorous discussion on the mathematical properties of the model.

## Results

### The cognitive state evolving as a quantum open system

We describe the *cognitive state* of the agent in a Hilbert space 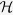, and we denote a state by |*ψ*〉. Let the state be definite, then the Schrödinger equation *d*|*ψ*(*t*)〉/*dt* = −*iH*|*ψ*(*t*)〉 formalizes its time evolution if the system is isolated, where *H* is the Hamiltonian (a Hermitian operator acting on 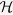) and *i*^2^ = −1. Nevertheless, in a general case we do not know the state, so the system has to be described by a mixed state or density matrix *ρ*. This *ρ* is a statistical mixture of pure states and formally it is a Hermitian, non-negative operator, whose trace is equal to one. The natural extension of the Schrödinger equation to density matrices is the von Neumann equation


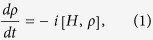


with [*H*, *ρ*] the commutator *Hρ* − *ρH*.

Furthermore, we consider that the best description for the ‘mind’ of an agent involved in a decision-making process is not as an isolated system, but one subject to *some* interaction with the environment. Therefore, its evolution is not given by the simple von Neumann equation.

Let our system of interest be a composite of two constituents: *M* and *E*, mind and environment. Due to the whole system (mind *and* environment) being isolated, *ρ*_*M+E*_ evolves according to the dynamics given in Eq. (1) by definition. From this, we can focus specifically on the state of *M* if we take partial trace over the Hilbert space of *E* such that the subsystem of the mind is *ρ*_*M*_ = Tr_*E*_(*ρ*_*M*+*E*_)^34^. Henceforth we drop the subindex *M* when referring to the state of the mind.

In order to know the equation of motion of *ρ*, we should take partial trace in Eq. (1), which is generally impossible. However, under the assumption of Markovianity (the evolution 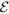 can be factorized as 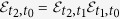 given a sequence of instants *t*_0_, *t*_1_, *t*_2_) one can find the most general form of this time evolution based on a time local master equation 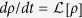, with 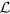 a differential superoperator (it acts over operators) called Lindbladian, embedding the standard form in which any Markovian master equation can be considered, and given by the Lindblad-Kossakowski equation^35^ such that


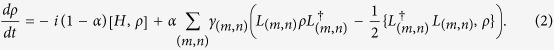


Here, *H* is the Hamiltonian of the subsystem of interest (in our case, the ‘mind’ of the decision-maker), the matrix with elements *γ*_(*m*, *n*)_ is positive semidefinite, *L*_(*m*, *n*)_ is a set of linear operators, and 
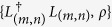
 denotes the anticommutator 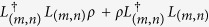. The meaning of the subindices (*m*, *n*) shall become clear later.

**Figure 1:**
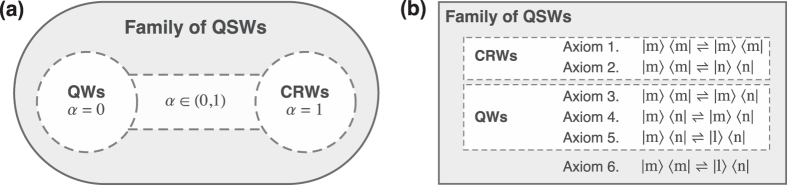
Quantum stochastic walks. (**a**) Venn diagram showing the relationship between quantum walks (QWs–unitary evolution defined by the von Neumann equation) and classical random walks (CRWs–irreversible dynamics in a master equation) as two limiting cases within the family of quantum stochastic walks (QSWs) which includes more general probability distributions. We make use of the subset of QSWs interpolating between both cases through the parameter *α* ∈ [0, 1]. (**b**) Axiomatic construction of QSWs from an underlying graph. Each element of the connectivity matrix of the network corresponds to an allowed transition in the dynamical process. CRWs are defined from Axioms 1-2, QWs from Axioms 3-5, and Axiom 6 is only associated to those QSWs with no counterpart as just CRWs or QWs for their formalization. See Whitfield *et al.*^36^ for further reading.

The second part in the master equation Eq. (2) contains the dissipative term responsible for the irreversibility in the decision-making process, weighted by the coefficient *α* such that the parameter *α* ∈ [0, 1] interpolates between the von Neumann evolution (*α* = 0) and the completely dissipative dynamics (*α* = 1). The section Methods covers the basics required to reach this formulation. See also Fig. 1 for an axiomatic construction of the quantum stochastic walks.

### From the tree to the network

A usual feature of many standard models for the analysis of decision-making problems is their representation as a graph with characteristics of a directed tree. We can understand such models as a root node 0 connected to each possible state of the world Ω ∈ *W* that the decision-maker can face. For each of this possible states, there is a set of weighted edges linking each state of the world Ω to the actions *i* ∈ *S* that the agent can take. These are nested models implying a sequential structure in the cognitive process: the agent is supposed to first form her (possibly own) beliefs about the (distribution of) states of the world and then optimize her action choice as a response to this information.

Our work departs from this standard setting and proposes a model where a richer networked structure of the decision-making mechanism represents an incessant flow of the agent’s response-probabilities conditioned on the topology of the problem. We propose that the decision-making process is a combination of the comparison of utilities taking place *simultaneously* with the elicitacion of beliefs and therefore removing the nested structure. The process extends over an interval of time and due to the dissipative dynamics we compute the unique stationary distribution of random walkers defining the behavioral choice-probabilities.

An appropriate definition of the so-called dissipators–operators *L*_(*m*, *n*)_ in Eq. (2)–allows the quantum formalism to also contain any classical random walk. The possible moves that the walker can make from each node can be described by a network, such that each node represents observable states of the system, and the edges account for the allowed transitions. This in turn relates the transition matrix defining the dynamics of the stochastic process to the structure of an underlying network. We prove in the Methods section how the dissipators lead to a unique stationary solution if they are defined as *L*_(*m*, *n*)_ = |*m*〉〈*n|*, with *γ*_(*m*, *n*)_ = *c*_*mn*_ being *c*_*mn*_ the entries of a *cognitive matrix C*(*λ*, *φ*) formalized as the linear combination of two matrices, Π(*λ*) and *B*, associated to the profitability comparison between alternatives and the formation of beliefs, respectively. The parameters *λ* and *φ* become meaningful in the next section, together with the definition of *C*(*λ*, *φ*) in Eq. (4).

### The cognitive matrix

As the starting point for defining the *cognitive matrix* in our model, we consider one of the most basic yet meaningful formulations of probabilistic choice theory: Luce’s choice axiom^37^,^38^,^39^. In this framework, given a choice-set *S* containing the available alternatives, the system of choice probabilities is defined by 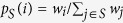, for every *i* ∈ *S*, with *w*_*i*_ being a scalar measure of some salient properties of the alternatives: a *weight* of each element within the set of available options.

A natural parametrization for the salience of each alternative *i* ∈ *S* is to define *w*_*i*_ = *u*(*i*|Ω)^*λ*^, where *u*(*i*|Ω) relates to the payoff the decision-maker obtains from taking action *i* if the state of the world is Ω. Because the terms *u*(·|·) have to be non-negative, situations with negative payoffs can be included after a monotonic transformation, the standard procedure in discrete choice theory. The exponent *λ* ∈ [0, ∞) measures the agent’s ability to discriminate the profitability among the different options. When *λ* = 0, each element *i* ∈ *S* has the same probability of being chosen (1/*N*_*S*_ with *N*_*S*_ the cardinality of the set *S*), and when *λ* → ∞ only the dominant alternative is chosen. If there is more than one option with the same maximum valuation, then the probability of an option being chosen is uniform within the restricted subset of the most preferred ones.

We now build the aforementioned matrix Π(*λ*) relying on the response probabilities *p*_*S*_(*i*) already defined. Let the connected components of the graph be in a bijection with the set of states of the world *W* such that each Ω ∈ *W* is related to one and only one connected component. The number of nodes *K*_Ω_ in the connected component associated to each possible state of the world Ω is the size of the corresponding action set. Let *n*_*i*_(Ω) be the node representing the event of the decision-maker taking action *i* when considering the state of the world is Ω. Then, every node *n*_*i*_(Ω) has *K*_Ω_ incoming flows of walkers, one from each of the other nodes *n*_*j*_(Ω) (*j* ≠ *i*) and one self-edge. These links are edges weighted in the spirit of Luce’s choice axiom,


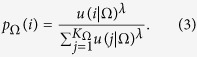


**Figure 2:**
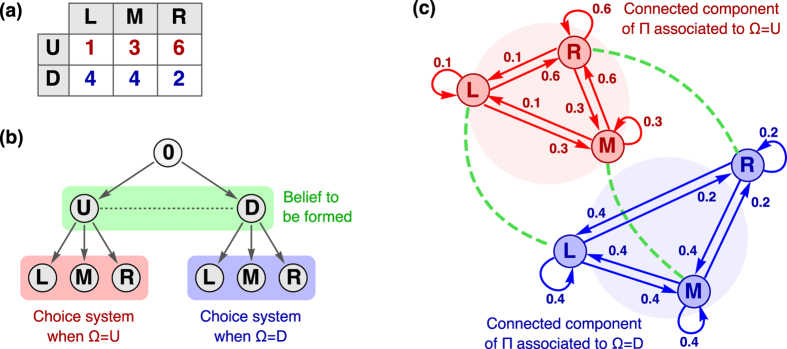
From the tree to the network. A first example step by step. We define an arbitrary toy-problem in which a decision-maker (the column-agent) chooses one option among *S* = {*L*, *M*, *R*}, with the payoff being a function of the state of the world Ω ∈ *W* = {*U*, *D*}. The numbers in panel (**a**) represent the profit for the column-agent given her choice of action and the state of the world that is realized, in the form of a payoff matrix. In panel (**b**) we show the normative representation of the sequential decision-making process as a tree. In this setting, the column-agent first makes her own belief about the state of the world and then, she optimizes her action as a response to her belief. In panel (**c**) we model the same problem with a networked topology. The numbers in the links represent the entries {*π*_*ij*_} of the matrix Π(*λ*). They are weighted according to Eq. (3), with *λ* = 1 and using the information in panel (**a**). These two connected components define the dynamical comparison between alternatives, for each possible state of the world. This process happens simultaneously with the formation of beliefs through the matrix *B* (green connections), as stated in Eq. (4). For basic illustrative purposes, we do not need to specify if the state of the world is a random variable, or the choice of a row-player whose payoff rule is unknown for the column-player. We elaborate further on this issue and its influence on the definition of the matrix *B* in the main text.

Note that every node *n*_*i*_(Ω) has *K*_Ω_ outgoing edges *e*_Ω_(*i*, *j*), generally with *K*_Ω_ different weights *p*_Ω_(*j*). See Fig. 2 for a graphical example deriving the matrix Π(*λ*) from the sequential tree.

We can define Π(*λ*) as a transition matrix where every entry *π*_*ij*_(Ω) is the probability that a random walker switches from action *i* to *j* for a given state of the world Ω. The navigation of random walkers along the network described by Π(*λ*) accounts for the comparison between alternatives for each given state of the world.

The decision-maker faces simultaneously another cognitive activity: the formation of her beliefs about the state of the world (either a forecast on some external random event, or a prediction on the behavior of an interacting agent). We model this process through the definition of the matrix *B* such that its entries connect nodes of the form *a*_*i*_(Ω_*k*_) to those of the form *a*_*i*_(Ω_*l*_). Thus, *B* allows the walker to introduce a *change of belief* about the state of the world in the cognitive process by jumping from one connected component associated to a particular state of the world Ω_*k*_ ∈ *W* to the connected component associated to another one Ω_*l*_ ∈ *W*, while keeping the action *i* fixed.

We denote the cognitive matrix by *C*(*λ*, *φ*), which is defined as


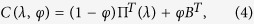


where *φ* ∈ (0, 1) is a parameter assessing the relevance of the formation of beliefs during the decision-making process. The superscript ^*T*^ denotes the transpose matrix. We discuss the reason for obtaining *C*(*λ*, *φ*) after the transposition of the transition matrix in the Methods section. Combining Π(*λ*) with *B* is crucial for the dynamics of the process: *B* establishes connections between the *N*_*W*_ (originally disjoint) connected components described by Π(*λ*). Therefore, *C*(*λ*, *φ*) describes a weighted (and oriented) graph with only one connected component which contains now *N*_*W*_ strongly connected components, one per each possible state of the world.

Typically, we may consider risky or uncertain situations to be objective if a random move has to be realized (lotteries), subjective if the agent has to evaluate probabilities based on her own judgment, or strategic if there is a game-theoretic interaction with hidden or simultaneous move of the opponents. As a consequence, there is a certain degree of arbitrariness in the way we can define the entries {*b*_*kl*_} for the matrix of belief formation, as long as its linear combination with Π(*λ*) guarantees existence and uniqueness of the stationary distribution ρ*. This is satisfied when the cognitive matrix fulfills the Perron-Frobenius theorem, *i.e.*, *C*(*λ*, *φ*) is irreducible and aperiodic^40^.

In the following part of the paper, we propose a definition for the matrix *B* in line with the standard models for strategic decision-making. Nevertheless, even if there is no particular information given by the problem, one can always define *b*_*kl*_(*i*) = 1/(*N*_*W*_ − 1), with *N*_*W*_ being the cardinality of the set *W*. This ‘homogenous’ law for the change of beliefs is reminiscent of the long-distance hopping matrix which has been fruitfully exploited in the study of ranking problems through quantum navigation of networks^41^,^42^.

### Analyzing the Prisoner’s Dilemma

The Prisoner’s Dilemma is widely considered to be the cognitive and game-theoretical task equivalent to the harmonic oscillator in physics: a well-defined problem with quite a simple formulation but still rich possibilities for both experimental and theoretical exploration, which qualifies this problem as the first system for which a new model should provide consistent explanation as a benchmark case study.

The symmetric Prisoner’s Dilemma is a game involving two players, *A* and *B*. They can choose among two actions: cooperate (*C*) or defect (*D*). Considering the game in its normal form, it is defined by the following payoff matrix,


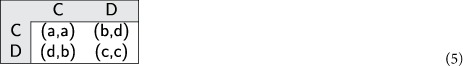


where *d* > *a* > *c* > *b* implies mutual cooperation is the Pareto optimal situation (maximum social payoff). In standard game theory, defection is the dominant strategy for both players, so mutual defection is the Nash equilibrium of this game (strategy profile stable against unilateral deviations), which is not an efficient outcome when compared against mutual cooperation. The rational prediction for the play of this game is the choice of defection as action, together with the expectation (belief) of facing also defection from the opponent, even though a fraction of cooperation usually appears when humans play this game^43^.

On the one hand, deviations from the purely rational (Nash) equilibrium in games can actually be modelled with classical probability theory if we consider stochastic choice-making with a finite value of the ‘rationality exponent’ analogous to the parameter *λ* already defined in Eq. (3)–see, *e.g.*, the concept of quantal response equilibrium^44^–but on the other hand, deeper empirical findings challenge the validity of the axioms of classical probability theory at their fundamental level: experiments with the Prisoner’s Dilemma game can also be used to show how the Sure Thing Principle (a direct consequence from the law of total probability in the classical framework) is violated^45^.

These two effects together lead Pothos *et al.*^46^ to formulate a quantum walk outperforming the predictions from the classical model, concluding that “human cognition can and should be modelled within a probabilistic framework, but classical probability theory is too restrictive to fully describe human cognition”. Their model incorporates a unitary evolution (QW) originated from a Hamiltonian operator implementing cognitive dissonances. Nevertheless, the unitary evolution lacks stationary solutions unless a stopping time is exogenously incorporated into the process, which raises also a fundamental concern about how to apply the class of models using QWs both from a conceptual and a practical standpoint^47^. We show later how the present model of QSWs accounts for these violations of the Sure Thing Principle in a natural way and that it has a stationary solution.

Thus, it is our intention to show how the more general formulation of quantum stochastic walks (QSWs) interpolating between both extreme cases (CRW with *α* = 1, and QW with *α* = 0) is able to incorporate the positive aspects of both models such as the relaxing dynamics towards a well-defined stationary solution from the classical perspective, together with the possibility for coupling and interference between populations and coherences through the unitary evolution. Besides, we respect the standard usage of the parameter *λ* as an upper-bound in the optimality of the solution, while our networked definition of the problem introduces new effects which are not reachable with the traditional representations of decision-making trees that do not allow for simultaneous exploration through both spaces of preferences and beliefs in parallel.

### Fine tuning the model

**Figure 3:**
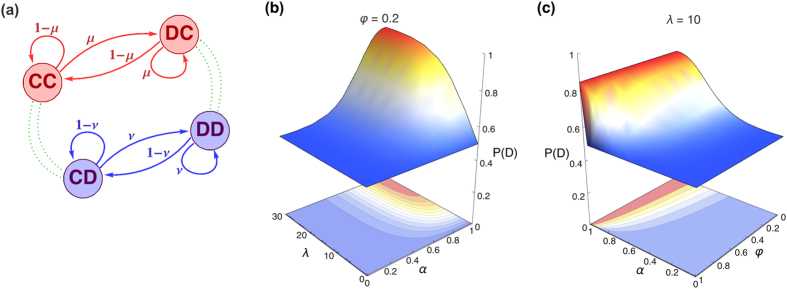
Modelling the Prisoner’s Dilemma (I). (**a**) Graphical representation of the connectivity pattern Π(*λ*_*a*_) defined in Eq. (6). The connected component in red color corresponds to the dynamic comparison of the two possible actions when expecting the opponent to cooperate, and the blue one represents the same process under the belief of receiving defection from the other player. With the only purpose of clarifying the structure of the matrix Π, the process of formation of beliefs is depicted just symbolically through the green links. (Note that we don’t show the self-edges). Because of the symmetry in the game, the components of the matrix *B*(*λ*_*b*_) can be related to those of Π, as we can see in Eq. (7) and the corresponding discussion in the main text. Panel (**b**) illustrates (for a fixed value of *φ* = 0.2) how the probability of choosing the action of defection increases with the rationality parameter *λ*. Panel (**c**) illustrates the probability of defection (for a fixed value of *λ* = 10) as a function of *α* and *φ*. See discussion in the main body of the text.

To illustrate the predictions of this class of models, we consider the payoff matrix used in the experiments conducted by Shafir and Tversky^45^, *a* = 75, *b* = 25, *c* = 30, and *d* = 85. From the point of view of any of the two players (this game is symmetric), the following identification for the action set and the possible states of the world is straightforward: *u*(*C*|*C*) = *a*, *u*(*C*|*D*) = *b*, *u*(*D*|*C*) = *d*, *u*(*D*|*D*) = *c*. We have a four-dimensional space of states 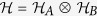 because two possible actions are associated to two possible states of the world. We choose the basis of the system spanning the space of states to be 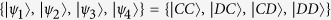. As an example, these pure states are defined such that 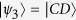 indicates the cognitive state in which the player chooses to execute the action *C* and holds the expectation of the opponent choosing *D*. The same definition holds for the other combinations. Following Eq. (3) and the definition of Π(*λ*) discussed in the introduction of the model, we write


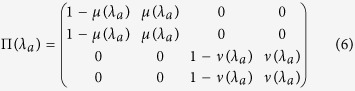


with 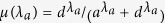, and 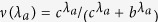. Then, for a given set of payoff values, the weights for the dynamics in the decision-making process are specific to the type of player given by the rationality parameter *λ*_*a*_, where the index *a* just indicates that the parameter *λ* is representative of the player comparing the actions. See panel (a) in Fig. 3 for its graphical representation as a network.

We are dealing with a situation of strategic uncertainty, so we define the matrix of formation of beliefs to be dependent on the payoff entries corresponding to the opponent, analogous to the definition of Π. In this case, the frequency with which a stochastic walker will jump from the belief associated to the state of the world *C* to the state of the world *D* is directly the estimation of how the other player is weighting her action *C* versus her action *D* during her deliberation on profitabilities, and then we write


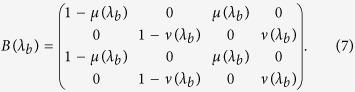


In a first step, *B* gets defined as a function of the rationality exponent associated to the second player whose action-set defines the set of possible states of the world faced by the first player. Because the game of the Prisoner’s Dilemma is symmetric, we can assume a common value *λ*_*a*_ = *λ*_*b*_ = *λ* which simplifies the model.

Combining these two connectivity patterns (do not forget the operation of matrix transposition) in the linear fashion defined in Eq. (4) finally determines the *cognitive matrix C*(*λ*, *φ*) given in Eq. (8),


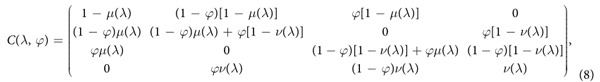


and modelling human behavior in Prisoner’s Dilemma games together with the Hamiltonian


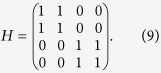


We use a simplified construction *H*_*ij*_ = 1 if the nodes are connected in Π(*λ*) and zero otherwise, in agreement with other applications of quantum rankings successfully explored in the literature about complex networks^42^. See a more thorough discussion on possible definitions of *H* in the corresponding section in Methods.

### Behavioral aspects

The cognitive matrix *C*(*λ*, *φ*) in Eq. (8) and the Hamiltonian operator in Eq. (9) define a three-parametric family of Lindbladian operators 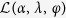 for the play of Prisoner’s Dilemma games. Since there exists a unique stationary solution ρ* for each evolution defined by the values of (*α*, *λ*, *φ*) as we prove in the Methods section, then we have defined a whole family of behaviors in the game which we analyze now as a function of the parameters.

#### *λ* as a measure of bounded rationality

We should consider the level of rationality in the play in comparison to the Nash equilibrium (*DD*), the reference of a rational outcome in the game. When we introduce *λ* in the model in Eq. (3), this parameter is a monotonic measure of the ability to discriminate between the profitability of the different options, and as such it has also a strictly monotonic influence on the level of rationality in the equilibrium predictions: the higher the *λ*, *cet. par.*, the higher is the probability of choosing the dominant action. See Fig. 3-Panel (b).

In our model, the parameter *λ* really plays the role of upper-*binding* the level of rationality in the process and not just a point-prediction. It determines the maximum probability of playing defection, while for a given *λ* different probability outcomes are achieved depending on the tradeoff between *α* and *φ*. See in Figs 3-Panel (c) and 4-Panel (a) how the weight on the belief formation in the dynamical process shapes the smoothness/steepness of the transition from pure randomization to the bounded level of rationality as a function of *α*, with the behavior getting closer to the allowed maximum when the process becomes more classical (*α* → 1).

**Figure 4:**
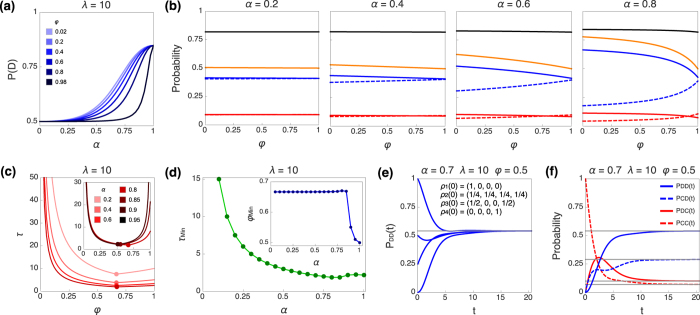
Modelling the Prisoner’s Dilemma (II). (**a**) Probability of defection (for a fixed value of *λ* = 10) as a function of *α*, and evaluated for different values of *φ* ∈ (0, 1) (see also Fig. 3-Panel (c) for complementary information). (**b**) Sequence of plots (also with *λ* = 10) for increasing values of *α* of the probabilities: *P*_Action=D_ = *P*_*DD*_ + *P*_*DC*_ (orange), *P*_Belief=D_ = *P*_*DD*_ + *P*_*CD*_ (black), *P*_*DD*_ (blue, solid), *P*_*CD*_ (blue, dashed), *P*_*DC*_ (red, solid), and *P*_*CC*_ (red, dashed), as a function of the parameter *φ*. (**c**) Relaxation time *τ*(*α*, *φ*) plotted as a function of *φ* for several values of *α*. For each curve, the dot indicates the minimum. The inset of the Panel corresponds to the highest values of *α*. (**d**) Minimum relaxation time *τ*_Min_(*α*) with inset of *φ*_Min_, both as a function of *α*. We define *φ*_Min_(*α*) as the value of *φ* minimizing the relaxation time for each value of *α*. (**e**) Temporal evolution of *P*_*DD*_(*t*) starting from four different initial conditions *ρ*(0). Legend’s notation *ρ*_*n*_(0) = (*ρ*_11_, *ρ*_22_, *ρ*_33_, *ρ*_44_) indicates the diagonal initial entries of the density matrix. We initialize the non-diagonal elements at zero value *ρ*_*ij*_(0) = 0 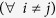 in the four cases. This plot illustrates the common convergence towards the stationary solution regardless of the initial values, as we show analytically in the Methods section. (**f**) Temporal evolution of the four components *P*_*ij*_(*t*) (*i*, *j* = *C*, *D*) for the initial condition *P*_*CC*_(0) = 1. Horizontal gray lines in panels (**e**,**f**) indicate the corresponding value of each component in the stationary solution. See further explanation of the graphs and the discussion regarding the behavioral aspects in the text.

We see in Fig. 4-Panel (b) how the finite limit in the level of rationality *λ* translates into a one-to-one correspondence with the expectation on the level of defection (black solid line), which remains basically constant and independent of the values of *α* and *φ*. Therefore, experimental results on belief elicitation can be used to adjust the numerical value of *λ*.

#### Believing the same to act different

This model based on the connected topology for the dynamical process combining simultaneously the formation of beliefs and the comparison of actions reveals an interesting effect: even for fixed values of *λ* (and then also fixed expectation on the rival’s move), it is possible to obtain different choice probabilities as a result of the different weights assigned to each of the two cognitive processes through *φ*.

We see in Fig. 4-Panel (b) how the probability of choosing defection as action (orange solid line) is decreasing on *φ* (for each possible value of *α*). This effect is very intuitive since higher *φ* implies less focusing on the discrimination between the profitability of own actions of the player. The dynamical process of decision-making incorporates this effect as a consequence of higher values of *λ* generating lower weights in the connections of the cognitive network *C*(*λ*, *φ*) inherited from the matrix Π(*λ*). This effect is hardly obtainable with standard models based on decision-making trees, since their sequential structure does not allow for the interaction between nodes belonging to different states of the world.

#### Relaxation time

By definition, *α* is the parameter interpolating between the unitary evolution (*α* = 0) which is a process of continuous oscillation without stationary solution, and the Markovian evolution (*α* = 1) which is dissipative and has a stationary solution. Thus, *α* is expected to play an important role in the determination of the relaxation time of our networked quantum stochastic model for decision-making. We denote it by *τ*, and we discuss its definition in the Methods section. As a cautious remark, let us say that the relaxation time of the dynamics may not always be the endogenously determined decision time of a subject on a given trial. The decision time will likely be a random variable with a distribution of stopping times. For further elaboration on this issue, see *e.g.*, Busemeyer *et al.*^30^ and Fuss and Navarro^48^.

Regarding the two cognitive parameters *λ* and *φ*, we observe no influence of *λ* on the magnitude of the relaxation time. We see in Fig. 4-Panel (c) how *τ* depends on *φ* with a clear minimum *τ*_Min_. The curve asymptotically diverges for *φ* → 0, while it remains finite for *φ* > 0 if *α* ∈ (0, 1], unless *α* = 1 and *φ* = 1, when *τ* diverges as well. As *α* approaches 1, *τ* vs. *φ* becomes very large for high values of *φ*, resulting in a U-shaped curve (inset of Fig. 4-Panel c). This comes from the tradeoff in the dynamics between the cognitive matrix *C*(*λ*, *φ*) and our choice of the Hamiltonian (Eq. 9). As the reader can see in the Methods sections, existence of the stationary solution requires the network to be connected such that no node is isolated, and the cognitive network represented by the matrix *C*(*λ*, *φ*) becomes disjoint if *φ* = 0, when there is no transition allowed between the components associated to the two states of the world. Thus, we can say that the presence of deliberation about the possible states of the world is crucial for the existence of a stationary solution, and therefore the process of construction of the belief is a key aspect in the convergence towards a stationary state.

In Fig. 4-Panel (d) we analyze *τ*_Min_ and *φ*_Min_ as a function of *α*. Considering *τ* as a function *τ*(*α*, *φ*), we implicitly define *φ*_Min_(*α*) as the value of *φ* for which *τ* is minimum, for each possible *α*. This figure clearly shows how the relaxation time remains finite for non-zero values of *α*, and decreasing the higher is the influence of the Markovian aspect of the dynamics. The abrupt step we observe in the relationship between *φ*_Min_ and the values of the parameter *α* is due to the breakdown of degeneracies in the spectrum of the Lindbladian superoperator at that point. Note that for a fully classical case 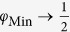, which would imply a fully homogeneous combination of Π(*λ*_*b*_) and *B*(*λ*_*b*_), in agreement with the symmetry of the Prisoner’s Dilemma problem and the choice of parametrization *λ*_*a*_ = *λ*_*b*_ = *λ*.

#### Stationary solution

We prove the existence and uniqueness of the stationary solution for our class of quantum stochastic walks for decision-making in the Methods section. Moreover, the solution is analytically defined and can be computed by exact diagonalization of the Lindbladian superoperator without the need for numerical simulations. Nevertheless, and only for illustrative purposes, we show the explicit evolution of the component *P*_*DD*_ for several initial conditions in Fig. 4-Panel (e), and the joint evolution of the four components starting from one extreme initial condition of full cooperation in Fig. 4-Panel (f).

Finally, let us emphasize the connection between the three parameters of the model and their observable counterparts. The bounded rationality parameter *λ* can be related to the result of the formation of beliefs about the opponent’s move. For a given *λ*, the choice probabilities in the space of actions and the relaxation time of the dynamical process are both governed by the pair (*α*, *φ*). Thus, we provide a model with three parameters to be estimated by the appropriate measurement of three observables. The presence of a distribution in stopping times can be proxied through a distribution of values for *τ* as a consequence of having a population of players with heterogeneous values of the parameters, such as different weights in the formation of expectations.

### Violation of the Sure Thing Principle

In the spirit of Pothos and Busemeyer^46^, we turn now to the application of this quantum model in explaining the so-called violations of the ‘Sure Thing’ Principle often observed in human behavior. This principle dates back to the work by Savage^49^ and can be understood as follows. Let a decision-maker decide between two options (*A* or *B*) when the actual state of the world (may it be the choice of an opponent, an objective lottery, or any other setting with uncertainty) is unknown, but the decision-maker knows that it can be either *X* or *Y*. Then, as a consequence of the (classical) law of total probability applied to modelling human behavior, if a decision-maker prefers *A* over *B* if the state of the world was known to be *X* and also prefers *A* over *B* if the state was known to be *Y*, she should also choose *A* when the state of the world is uknown because *A* is superior to *B* for every expectation on the realization of *X*/*Y*. Nevertheless, this principle was already refuted in an experiment by Tversky and Shafir^50^, an observation which has been regularly reproduced afterwards.

Busemeyer *et al.*^51^ and Pothos and Busemeyer^46^ provide a further review of empirical evidence on this issue and also show how quantum-inspired models can account for this effect, outperforming the classical ones. They explicitly compare models based on unitary evolution of the decision-making probabilities versus their Markovian counterparts. Despite of the (qualitative and quantitative) success of these quantum-like models, they are subject to the already mentioned criticism of lacking stationary solutions defined endogenously. We want to briefly show here how the stationary states of the quantum stochastic walks that we have defined in this paper can model the violations of the Savage’s Principle in a parsimonious manner. Furthermore, this effect is available only if the model is not restricted to its classical part (*α* = 1) but applied in its general way (0 < *α* < 1), emphasizing the synergies from combining both the quantum and the classical term in this dynamics.

**Figure 5:**
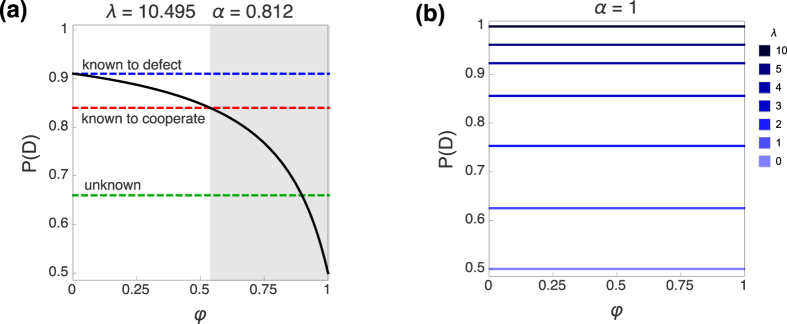
Violation of the Sure Thing Principle. (**a**) Model fit for the experimental results in Busemeyer *et al.*^51^. See discussion in the main text. (**b**) Probability of defection for different values of *λ* independent of *φ* when the model is restricted to the classical part (*α* = 1).

In order to make our case, we reproduce the experimental results in Busemeyer *et al.*^51^. The entries of the payoff matrix are *a* = 20, *b* = 5, *c* = 10, and *d* = 25. Their results show a defection rate of 91% when the subjects know their opponent will defect, and of 84% when they know the rival’s action is to cooperate. The Sure Thing Principle is violated in this experiment because the defection rate when the choice of the opponents is unknown drops to 66%. See model fit to this data in Fig. 5-Panel (a).

First, we consider the two defection rates when the state of the world is known, and use them to obtain the best fit of the model under the constraint *φ* = 0, because in these two situations the decision-maker does not need to allocate any effort to build an expectation about the rival’s move since it is fixed by default. The dynamics are solved numerically, and we choose the density matrices with diagonal elements 
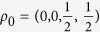
 and 
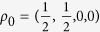
 as initial points for the two scenarios (the rival defects or cooperates) such that the system is confined to the subspace of each announced state of the world. We obtain the best fit for the parameter values *λ* = 10.495 and *α* = 0.812, yielding predictions of 0.911 and 0.839 for the two defection rates in the sure situations.

Second, we take these values for (*α*, *λ*) as fixed and study the impact of introducing uncertainty in the decision-making process. This is modelled by the parameter *φ* > 0, which means that the decision-maker has to assign some effort to the ‘guessing task’. We see in Panel (a) that the quantum stochastic walk naturally includes violations of the Sure Thing Principle in this setting when the weight of the matrix *B* in the dynamics becomes more relevant. We obtain 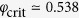 as the critical value of *φ* for which the predicted outcome for this experiment lies below the defection rate of 84%, and the value *φ*_exp_ = 0.898 models the experimental result of only 66% of defection in the uncertain situation. Finally, Fig. 5-Panel (b) illustrates how this effect is not available when only the classical term is considered (by fixing *α* = 1). It is straightforward to see how in such a classical case, the prediction (for any value of *λ*) is independent of the parameter *φ*. One can understand this by noticing that several type of transitions are not present in the dynamics when only the CRW applies (see Fig. 1-Panel (b) once again).

## Discussion

Understanding how us humans process the information that we retrieve from our environment and how this affects our ability to make decisions is of major relevance in the analysis of individuals’ behavior under circumstances of risk and uncertainty, and we consider that the interplay between quantum and classical random walks may be a promising attempt to incorporate human-like reasoning biases in the formulation of stochastic decision-making dynamics. Furthemore, the quantum nature of this algorithm does not imply any quantum functioning of the physical substrate (the *brain*) in which the decision-making process is embedded at all, in the same way that quantum navigation of networks for ranking their nodes does not require for any quantum hardware and outperforms the classical ranking techniques^42^.

We have proposed a new way to model the deliberation process undergoing any decision-making mechanism via the navigation of small cognitive networks with quantum walkers. Our class of models can extend the dynamic-stochastic theory of decision-making to the quantum domain, incorporating coherences in a random walk which occurs along an otherwise classical set of nodes. This hybrid dynamics defines a unique and stationary distribution for the stochastic behavior. In our illustrative example, we build the cognitive network to perform only the two cognitive operations required in the Prisoner’s Dilemma game: the comparison between the payoffs of one’s own actions, and the estimation of other players’ moves. Of course, the definition is able to contain the weighted combination of any finite number of tasks, with the linear coefficients representing the decision-maker’s allocation of relative efforts.

The application of these quantum stochastic walks on networks for decision-making shares the building blocks of the renowned decision field theory^52^, already formulated as a connectivist model^53^. We consider this is a promising avenue of research in order to bring the successful stochastic-dynamic family of cognitive models into the quantum domain. This generalization is a natural step given the latest evidence available^54^, especially the experiment by Busemeyer *et al.*^55^, designed to prove wrong the belief that quantum models fit better just because they are more complex.

## Methods

### Hilbert space and density matrix

For finite dimensional systems, a Hilbert space 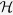 is simply a linear space endowed with a scalar product 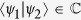. Its elements (or states) are denoted by 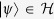. We consider only states with non-vanishing norm 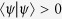. If the state of the system is 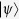 we say it is in a *pure state*.

The projector 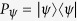, an operator acting on 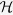 as 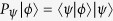, has a bijective relation with |*ψ*〉, so we can describe the state |*ψ*〉 in terms of *P*_*ψ*_.

A density matrix *ρ* is an operator acting on 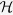, 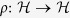 with the following properties: (*i*) it is Hermitian: *ρ*^†^ = *ρ*, (*ii*) it has trace one: Tr(*ρ*) = 1, and (*iii*) it is positive semi-definite: 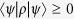 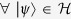.

Any *ρ* can be written as 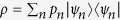, with 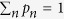. Notice that if *p*_1_ = 1 and *p*_*n*_ = 0 for *n* > 1, then 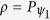, so *ρ* describes the pure state |*ψ*_1_〉. In general *p*_*n*_ > 0 ∀*n* and *ρ* is not a projector. In such a case, *ρ* describes a situation where we have some uncertainty about the state of the system with the probability of the system being in |*ψ*_*n*_〉 given by *p*_*n*_, and we say the system is in a *mixed state*.

### Relationship between quantum and classical random walks

A comprehensive approach to quantum stochastic dynamics (QSWs) can be achieved by considering the classical random walk in discrete time as the basic setup, grounding the path towards the more sophisticated quantum formulation. Here, we first define the CRW in discrete time, and later extend its formulation naturally to the continuous time domain. Second, we introduce the QW directly in continuous time and bring both of them together, supporting the formulation of Eq. (2) already stated.

Let us consider a classical random walk in its discrete time version for which there is a certain set of *N* possible states of the system. At each time step *t*, the system may transit to state *i* from state *j* according to the relations defined in a *N* × *N* transition matrix *T* = {*T*_*ij*_}. The state of the system is described by a vector *p*(*t*) ∈ Δ^Ν^ (Δ^Ν^ is the *N*-dimensional simplex, such that every component *p*_*i*_(*t*) ≥ 0 and 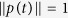, ∀*t*). If the state of the system at time *t* − 1 is *p*(*t* − 1), the state of the system at the following time step *t* is 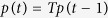 and then, if the initial condition is *p*(0), the state of the system after *t* steps is given by *p*(*t*) = *T*^*t*^*p*(0), where *T*^*t*^ is the *t*-th power of the transition matrix.

A standard microfoundation for this process comes when picturing the system evolution as the evolution of the distribution of a random walker hopping along a network composed of *N* nodes (one per each possible state) and defined such that its connectivity pattern *A* = {*a*_*ij*_} generates the dynamics in *T*. The *i*-th component of the state-vector *p*(*t*) accounts for the probability of the walker being found in node *i* at time *t*. Given two consecutive instants (*t* − 1, *t*), the distribution changes according to 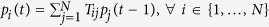. The edges of the network are defined such that *a*_*ij*_ represents a link from node *i* to node *j* (out-flow orientation), so it is straightforward to observe how the transition matrix *T* (which denotes transitions in the in-flow orientation) is related to the connectivity pattern *A* through the operation of matrix transposition (and a possible operation of connectivity normalization to preserve the total probability equal to one via the out-degree of the nodes 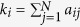 if the network is not weighted a priori). Because our cognitive matrix *C*(*λ*, *φ*) directly determines the stochastic evolution, the reader can now see why *T* = *C*(*λ*, *φ*) is defined from the transposed matrices Π^*T*^ and *B*^*T*^ in Eq. (4). These dynamics (and their continuous counterpart) tends to a unique stationary solution if the network is connected.

Provided the understanding of the discrete time transition process, the continuous time CRW reads as 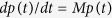, where 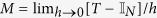, and 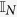 is the *N* × *N* identity matrix.

We now turn to the quantum case and introduce the *N* × *N* quantum density operator *ρ* playing the role analogous to the state-vector *p* in the classical case. The relationship between quantum and classical random walks is made through the occupation probabilities defined such that *ρ*_*ii*_ = 〈*i*|*ρ*|*i*〉 = *p*_*i*_. From the discussion above, it follows that the Markovian master equation can be written as 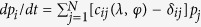 (*δ*_*ij*_ is the Kronecker symbol such that *δ*_*ij*_ = 1 only if *i* = *j*, and zero otherwise), and it can be shown how this walk can be quantized identifying *M*_*ij*_ = 〈*i*|*H*|*j*〉, with *H* being an Hermitian operator (the Hamiltonian) ensuring that *M* is a real matrix. *M* can be asymmetric for classical models, and in this more general case, asymmetries can be incorporated via the Lindblad operators. This approach takes the Schrödinger evolution as the building block, and depending on certain properties of the system the task of classical-to-quantum identification might not be straightforward^41^,^42^, but as Whitfield *et al.*^36^ show, both classical irreversibility and quantum coherence can be brought together applying the Markovian master equation for density matrices we introduced in Eq. (2) of the main body of the paper. Using the definition of the Lindbladian operators discussed in the main text, we directly obtain the classical part in the evolution of the diagonal terms (populations) of the density matrix given as 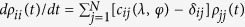.

### Defining the Hamiltonian

The Hamiltonian introduces the quantumness in the dynamics. For the case of undirected networks it is usual to define 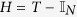. Nevertheless, because we deal with a walk along a weighted and directed graph, we simplify the definition to 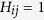 if the nodes are connected in Π(*λ*) and zero otherwise, in agreement with other applications of quantum rankings successfully explored in the literature on complex networks^42^. One may consider more intricate definitions of this operator like 
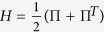
 as long as it remains symmetric when restricted to the real domain (*H* = *H*^*T*^), or Hermitian (*H* = *H*^†^) in general. Summarizing, the Hamiltonian couples diagonal and non-diagonal elements in the dynamics (known as coherence). The classical term is responsible for the exponential decayment of the non-diagonal elements of the density matrix over time and for the existence of a steady state.

### Existence and uniqueness of the stationary solution

In order to prove that the stationary solution for our class of models exists and is unique we draw upon Spohn’s 1977 theorem^56^:

*Given a Lindbladian evolution –*Eq. (2)–, the dynamic is relaxing (it tends to a stationary solution ρ* for any initial condition) if

- *the set* {*L*_(*m*, *n*)_} *is self-adjoint (this means the adjoint of L*_(*m*, *n*)_*, denoted by* 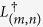
*, belongs to the set), and*
- *the only operator commuting with all the L*_(*m*, *n*)_ *is proportional to the identity.*

We show how our system fulfills both conditions.

#### Proof

The first condition is trivially satisfied. If *c*_*mn*_ ≠ 0, we have *L*_(*m*, *n*)_ = |*m*〉〈*n*|. Then 
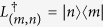
, which is equal to *L*_(*n*, *m*)_ if *c*_*mn*_ ≠ 0. Due to the definition of *C*(*λ*, *φ*), this holds unless *λ* → ∞, so our system follows the first condition of the theorem.

Second, we show that the only operator commuting with *L*_(*m*, *n*)_ is proportional to the identity. This is, if 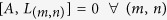, then 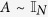. First, we consider a generic operator 
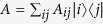
. We take the commmutator of *A* with 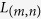, and we impose that it vanishes:


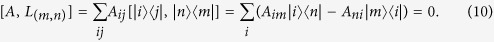


Computing the matrix elements of this commutator 〈*l*|[*A*, *L*_(*m*, *n*)_]|*k*〉,


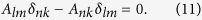


Taking *l* = *m* and *k* ≠ *n* we obtain *A*_*nk*_ = 0, and taking *l* ≠ *m* and *k* = *n*, then *A*_*lm*_ = 0. We have just shown that the matrix *A* is diagonal. In order to figure out how the diagonal elements are, we fix *m* = *m*_1_ and define 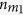 such that 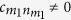. Now we consider 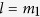, 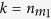 in Eq. (11), 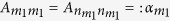.

In this way, we prove that the submatrix of *A* corresponding to node *m*_1_ and all the nodes directly linked to it is proportional to the identity matrix. By repeating the same procedure with *m* = *m*_2_ such that 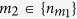 (this means *m*_2_ is linked to *m*_1_), we show that the submatrix related to *m*_2_ and the nodes linked to it is also proportional to the identity, but the proportionality constant must be the same as the one for the submatrix of nodes connected to *m*_1_, because *m*_2_ is linked to *m*_1_. As our network is connected, we eventually reach 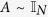 for *φ* ∈ (0, 1) in an iterative manner.

We have proven here that the second condition is also fulfilled, so the stationary solution of our system does exist and is unique.

We denote the stationary distribution by ρ*, and we give further details about its computation below.

### Vectorization of *ρ*

In order to solve the Lindblad-Kossakowski equation (Eq. 2 in the main text), we need to rewrite it as a matrix equation:


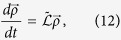


where 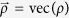 is the vector with *N*^2^ components vectorizing the density matrix *ρ*, (*i.e.*, a column vector formed by the columns of *ρ* arranged one after another), and 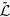 is the superoperator 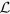 in its *N*^2^ × *N*^2^ matrix form. To that end, we insert the identity operator into the Lindblad-Kossakowski equation:


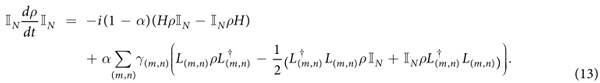


At this point we need to introduce the following tensor identity^57^,^58^, 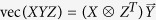, with *X*, *Y*, and *Z* being matrices, and 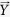 the aforementioned vectorization of *Y*. Then, we obtain in Eq. (13),


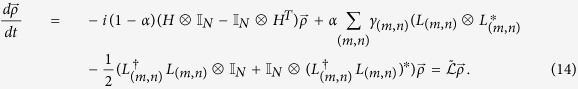


The formal solution of Eq. (14) for any given initial condition 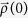 is 
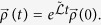
 Once we have vectorized the Lindblad-Kossakowski equation, we solve it by means of exact diagonalization of 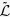.

### Stationary solution and relaxation time

The full spectrum of the Lindbladian 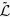 provides all the information about the system. However, a lot can be known by partial knowledge of it. It can be shown that any 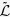 fulfilling the conditions of the theorem above can be decomposed as a direct sum of Jordan forms^35^. There exists a matrix *S* such that 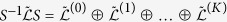, where 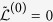, and the others are


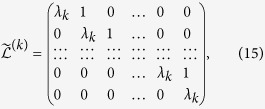


with 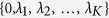 being the eigenvalues of 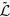.

The evolution superoperator becomes 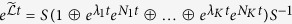, with *N*_*k*_ being nilpotent matrices. As Re(*λ*_*k*_) < 0 because of the existence and uniqueness theorem, the only term surviving for *t* → ∞ is the one corresponding to 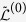. Hence, the eigenvector associated to the eigenvalue 0 is the vectorized form of the stationary solution ρ*. Besides, if we order the eigenvalues such that 0 > Re(*λ*_1_) > Re(*λ*_2_) > … > Re(*λ*_*K*_), the relaxation time is given by *τ* = −1/Re(*λ*_1_). This is the definition we use throughout the paper.

## Additional Information

**How to cite this article**: Martínez-Martínez, I. and Sánchez-Burillo, E. Quantum stochastic walks on networks for decision-making. *Sci. Rep.* **6**, 23812; doi: 10.1038/srep23812 (2016).
